# A novel variant of *PLA2G6* gene related early-onset parkinsonism: a case report and literature review

**DOI:** 10.3389/fneur.2024.1349861

**Published:** 2024-04-18

**Authors:** Dapeng Cai, Haohao Wu, Baogang Huang, Weiwei Xiao, Kang Du

**Affiliations:** Department of Neurology, Qujing First People's Hospital, Qujing, Yunnan, China

**Keywords:** *PLA2G6*, early-onset parkinsonism, cerebellar atrophy, hot spot mutation, heterogeneity

## Abstract

This study reported a case of early-onset parkinsonism associated with a novel variant of the PLA2G6 gene. The boy first started showing symptoms at the age of 11, with gait instability and frequent falls. As the disease progressed, his gait instability worsened, and he developed difficulties with swallowing and speaking, although there was no apparent decline in cognitive function. An MRI of the head revealed significant atrophy of the cerebellum. The initial diagnosis for the boy was early-onset parkinsonism, classified as Hoehn-Yahr grade 5.Genomic sequencing of the patient indicated that he had compound heterozygous variations in the PLA2G6 gene: c.1454G>A (p.Gly485Glu) and c.991G>T (p.Asp331Tyr). Pedigree analysis revealed that his younger brother also carried the same variant, albeit with milder symptoms. The patient's unaffected mother was found to be a carrier of the c.991G>T variant. Additionally, this study reviewed 62 unrelated families with PLA2G6 gene-related early-onset parkinsonism. The analysis showed a higher proportion of female probands, with a mean age of onset of ~23.0 years. Primary symptoms were predominantly bradykinesia and psychosis, with tremors being relatively rare. Cerebellar atrophy was observed in 41 patients (66.1%). Among the reported mutations, the most common mutation was c.991G>T, presenting in 21 families (33.9%), followed by c.2222G>A in eight families (12.9%). Other mutations were less common. Notably, the c.991G>T mutation was exclusive to Chinese families and was a prevalent mutation among this population. The initial symptoms varied significantly among patients with different mutations.

## 1 Introduction

Early-onset parkinsonism (EOP) is a neurodegenerative disease related to genetic factors. PLA2G6 gene mutation is considered to be one of the pathogenic genes involved in the development of EOP (1). Autosomal recessive EOP caused by mutations in the PLA2G6 gene is called PLA2G6-associated Neurodegeneration (PLAN) (2, 3). These include Infantile neuroaxonal dystrophy (INAD), Atypical neuroaxonal dystrophy (ANAD), and EOP (4). In this study, a case of EOP caused by a novel PLA2G6 gene mutation was reported, and previous reports of EOP related to this gene were reviewed.

## 2 Case presentation

A 22-year-old male patient was admitted to the hospital due to gait abnormality and frequent falls. The patient developed the above symptoms at the age of 11, and his motor development was slightly worse than that of his peers. After that, the patient's gait instability was aggravated, dysphagia and dysarthria gradually appeared, without obvious cognitive decline, and no special diagnosis and treatment were given. The patient's anomalies of gait and weakness of extremities were further aggravated, manifested as frequent falls, requiring bed rest or wheelchair. The patient's articulation disorder and deglutition disorders were aggravated compared with the previous ones, and the cognitive decline was presented, but he could still communicate normally with his family members. The proband's father died of trauma. Prior to his death, he denied the anomalies of gait, muscle weakness and other symptoms. The proband's younger brother began to have gait instability at the age of 11, and his motor development since childhood was slightly worse than that of his peers. At the age of 20, he can still walk normally, live independently, but his muscle tension is symmetrically increased. The proband's mother had a head trembling a few months ago, without other special discomforts. The patient's cranial magnetic resonance imaging (MRI) examination in September 2022 revealed cerebellar atrophy. The diagnosis of Parkinson's syndrome was made, and the Hoehn-Yahr grade was 5.

The nervous system physical examination revealed that the patient had normal vital signs, clear mind, but had severe dysarthria. The orientation of time, character and place was normal, the calculation and comprehension were decreased. The cranial nerves examination did not reveal any abnormalities. The distal and proximal muscle strength of both upper limbs was grade 3, the distal and proximal muscle strength of both lower limbs was grade 2, the symmetry of muscle tension of both upper limbs was increased, the muscle tension of both lower limbs was decreased, the tendon reflex of both upper limbs was brisk, the tendon reflex of both lower limbs was absent, the Rossolimo's sign of both upper limbs was positive, the pathological sign of both lower limbs was negative, the meningeal irritation sign was negative, and the patient had no sensory abnormalities and ataxia signs in the physical examination. Wide base gait, slow movement, reduced swing arms of both hands, and unilateral assistance during walking. The patient could not cooperate to complete the bilateral finger-nose test, heel-knee-tibia test, and pull-back test.

**Figure 1:**
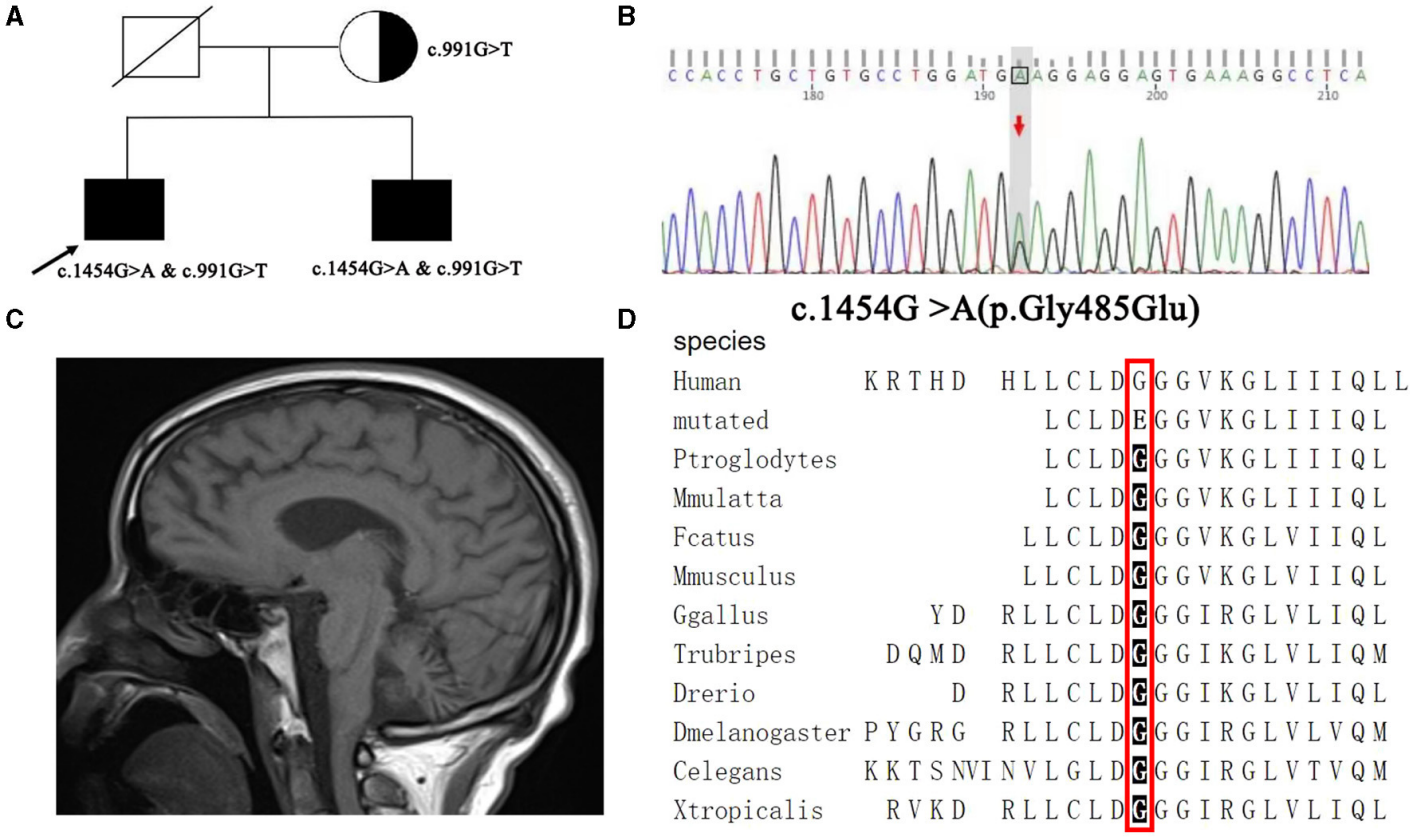
**(A)** Family diagram of the proband; **(B)** Another complex heterozygous variation of proband c.1454G>A with Sanger sequencing; **(C)** Cerebellar atrophy can be seen in the sagittal T1WI phase of the proband's head MRI; **(D)** The conservative analysis of this variant suggests that it is highly conservative.

The results of auxiliary examination suggest that: there were no abnormalities in hematuria, stool routine, biochemical indicators, homocysteine, ceruloplasmin, hepatitis, syphilis, HIV, coagulation function, autoimmune antibody spectrum, and cardiac ultrasound. MRI plain scan and contrast-enhancement of the head indicated brain atrophy, especially in the bilateral cerebellar hemispheres (Figure 1C).

The patient's whole genome sequencing suggested that the PLA2G6 gene compound heterozygous variants c.1454G > A (p.Gly485Glu), c.991G > T (p.Asp331Tyr) (Figures 1A, B). The results of pedigree verification suggested that the proband's brother was consistent with the proband's results. The proband's mother was a asymptomatic carrier of the variant of PLA2G6 gene c.991G > T. The proband's father failed to perform pedigree verification due to unexpected death, which was consistent with the role of family co-segregation. The variant of c.991G > T has been previously reported as a pathogenic variation (5), however, the variant of c.1454G > A has not been reported. The Mutation Taster software predicted it as a pathogenic variation, and the probability was 0.9999; this variant was not found in ExAC and Thousand Human Genome Database, and the conservation analysis suggested that it was highly conserved (Figure 1D).

The patient was finally diagnosed with PLA2G6 gene-related early-onset Parkinson's syndrome. The patient was treated with madopa 62.5 mg tid gradually increased to 125 mg tid orally. After 2 months of follow-up, the patient's gait abnormality was slightly improved compared with the previous one, and the disease did not progress significantly.

## 3 Literature review

In this study, the keywords “PLA2G6,” “parkinsonism,” and “Parkinson” were searched through “Pubmed,” “Wanfang Medicine,” and “China National Knowledge Infrastructure” databases. The literature of PLA2G6 gene-related EOP patients reported in Chinese and English was reviewed. A total of 62 families, including 30 Chinese families, were reviewed, and the clinical and genetic characteristics of the probands in all families were summarized (Table 1).

**Table 1:** A retrospective analysis of *PLA2G6* gene-related EOP literature has been reported.

**Family number**	**Family source**	**Sex**	**Age of onset (years)**	**Initial symptom**	**Clinical manifestation**	**Head MRI**	**Treatment**	**Variant 1**	**Variant 2**	**Author/year**
1	English	F	26	Cognitive decline, movement disorder, clumsiness, frequent falls, trembling, and dysarthria	Cognitive decline, dystonia, movement disorder, and rigidity	Normal	Levodopa/effective	c.2222G>A	c.2222G > A	Paisan-Ruiz et al. 2009 (6)
2	English	F	18	Cognitive decline, dysphoria, and movement disorder	Gait disorder, dystonia, and mental disorder	Normal	Levodopa/effective	c.2239C > T	c.2239C > T	Paisan-Ruiz et al. 2009 (6)
3	Iranian	M	25	Gait disorder	Gait disorder, dystonia, and cognitive decline	Normal	NA	c.1894C>T	c.1894C>T	Sina et al. 2009 (7)
4	Japanese	F	20	Resting tremor and gait instability	Resting tremor, movement disorder, rigidity, anomalies of posture, gait disorder, depression, mental disorder, and cognitive decline	Normal	NA	c.216C>A	c.1904G>A	Yoshino et al. 2010 (8)
5	Japanese	M	25	Movement disorder and gait disorder	Resting tremor, movement disorder, rigidity, anomalies of posture, gait disorder, and cognitive decline	Normal	NA	c.1354C>T	c.1904G>A	Yoshino et al. 2010 (8)
6	French	F	18	Depression	Dystonia, depression, dyspraxia, and frequent falls	Cerebellar atrophy.	NA	c.4C>A	Del Ex 3	Bower et al. 2011 (9)
7	Chinese	M	37	Gait disorder	Resting tremor, movement disorder, rigidity, anomalies of posture, and gait disorder	Normal	NA	c.991G>T	c.991G>T	Shi et al. 2011 (5)
8	Scandinavian	F	22	Depression	Resting tremor, movement disorder, rigidity, anomalies of posture, gait disorder, depression, mental and behavioral disorders, cognitive decline, and dystonia	Normal	NA	c.G238A	c.G238A	Agarwal et al. 2012 (10)
9	Chinese	F	19	Movement disorder and gait disorder	Resting tremor, severe muscle rigidity, movement disorder, defective coordination, trembling, mental and behavioral disorders, and dysarthria	Cerebellar atrophy.	Levodopa/effective	c.991G>T	c.1077G>A	Lu et al. 2012 (11)
10	Chinese	F	8	Movement disorder and gait disorder	Resting tremor, severe muscle rigidity, movement disorder, defective coordination, mental and behavioral disorders and dysarthria	Cerebellar atrophy.	Levodopa/effective	c.991G>T	c.1078G>A	Lu et al. 2012 (11)
11	Chinese	F	30	Movement disorders	Severe muscle rigidity, movement disorder, defective coordination, and mental and behavioral disorders	Normal	Levodopa/effective	c.991G>T	c.991G>T	Lu et al. 2012 (11)
12	English	F	18	Movement disorder and gait disorder	Defective coordination, dysarthria, dysphagia, rigidity, and movement disorder	Cerebellar atrophy.	NA	c.109C>T	c.1078_3C>A	Paisán-Ruiz et al. 2012 (12)
13	Greek	F	22	Dysphoria and visual illusion	Dysphoria, trembling, movement disorder, and frequent falls	Cerebellar atrophy.	Levodopa/effective	c.1715C>T	c.1715C>T	Paisán-Ruiz et al. 2012 (12)
14	The white race	F	3	Gait instability	Dystonia, muscle rigidity, gait instability, and dysphoria	Cerebellar atrophy.	NA	c.2370T>G	c.691G>C	Illingworth et al. 2014 (13)
15	American	F	25	Trembling, gait disorder, and depression	Dystonia, dysphagia, depression, dysphoria and mental disorder	Cerebellar atrophy.	Levodopa/Invalid	c.2222G>A	c.2222G>A	Virmani et al. 2014 (14)
16	Italian	F	27	Gait disorder, movement disorder, dysarthria, and dysphoria	Movement disorder, frequent falls, rigidity, dystonia, dysphoria, mental disorder, and cognitive impairment	Normal	Levodopa/effective	c.1547C>T	c.1547C>T	Malaguti et al. 2015 (15)
17	Chinese	M	36	Gait disorder	Gait disorder, dystonia, movement disorder, and rigidity	Normal	Levodopa/effective	c.991G>T	c.991G>T	Xie et al. 2015 (16)
18	Chinese	M	36	Static tremor	Resting tremor, dystonia, movement disorder, and rigidity	Normal	Levodopa/effective	c.991G>T	c.991G>T	Xie et al. 2015 (16)
19	Koreans	F	22	Gait instability and dysarthria	Dystonia, disorder, frequent falls, and dysarthria	Normal	Levodopa/effective	c.1039G>A	c.1670C>T	Kim et al. 2015 (17)
20	Turks	F	27	Movement disorder	Dystonia, Resting tremor, movement disorder, rigidity, anomalies of posture, gait disorder, movement disorder, depression, mental disorder, and cognitive decline	Normal	NA	c.2239C>T	c.2239C>T	Giri et al. 2016 (18)
21	Arab	F	26	Gait disorder	Dystonia, depression, and movement disorder	Normal	NA	c.2222G>A	c.2222G>A	Bohlega et al. 2016 (19)
22	German	F	21	Gait disorder	Movement disorder and gait disorder	Cerebellar atrophy.	Levodopa/effective	c.610-1G>T	c.1627C>T	Klein et al. 2016 (20)
23	Indian	M	9	Cognitive decline and trembling	Dystonia and defective coordination	Cerebellar atrophy.	Levodopa/effective	c.1946G>A	c.1946G>A	Kapoor et al. 2016 (21)
24	Saudi Arabia	F	26	Depression and movement disorder	Resting tremor, movement disorder, rigidity, anomalies of posture, gait disorder, depression, mental and behavioral disorders, and cognitive decline	Normal	NA	c.2222G>A	c.2222G>A	Bohlega et al. 2016 (19)
25	Chinese	M	27	Gait disorder	Resting tremor, movement disorder, rigidity, anomalies of posture, and gait disorder	Cerebellar atrophy.	Levodopa/effective	c.668C>T	c.1915G>A	Chen et al. 2018 (22)
26	Chinese	M	29	Gait disorder	Anomalies of posture, gait disorder, movement disorder, trembling, dysarthria, and dystonia	Cerebellar atrophy.	NA	c.991G>T	c.1982C>T	Chen et al. 2018 (22)
27	Chinese	M	30	Gait disorder and frequent falls	Rigidity, movement disorder, frequent falls, cognitive decline, and gait disorder	Cerebellar atrophy.	Levodopa/effective	c. 991G>T	c.1472+1G>A	Shen et al. 2019 (23)
28	Kuwaitians	F	17	Mental disorder, depression, and dysphoria	Mental disorder, depression, dysphoria, rigidity, Dystonia, gait instability, and frequent falls	Cerebellar atrophy.	Levodopa/effective	c.2215G>C	c.2215G>C	Kamel et al. 2019 (24)
29	Chinese	M	17	Dystonia, dysarthria, and cognitive decline	Frequent falls, mental disorder, dysarthria, dystonia, and epilepsy	Cerebellar atrophy.	NA	c.991G>T	c.238G>A	Ma et al. 2019 (25)
30	Indian	F	3	Gait disorder and cognitive decline	Rigidity, slow development, gait instability, and cognitive decline	Cerebellar atrophy.	NA	c.1798C>T	c.2357C>T	Jain et al. 2019 (26)
31	Chinese	F	30	Movement disorder	Resting tremor, movement disorder, rigidity, anomalies of posture, depression, mental disorder, cognitive decline, and gait disorder	Normal	Levodopa/effective	c.991G>T	c.991G>T	Chu et al. 2020 (27)
32	Chinese	F	26	Clumsiness	Resting tremor, movement disorder, rigidity, anomalies of posture, depression, mental and behavioral disorders, cognitive decline, and gait disorder	Normal	Levodopa/effective	c.991G>T	c.991G>T	Chu et al. 2020 (27)
33	Chinese	M	31	Depression	Dysarthria, movement disorder, and dystonia	Cerebellar atrophy.	Levodopa/effective	c.991G>T	c.1077G>A	Chu et al. 2020 (27)
34	Chinese	F	34	Resting tremor and fatigue	Resting tremor, movement disorder, and memory decline	Normal	Levodopa/effective	c.1321T>C	c.856delT	Gao et al. 2020 (28)
35	Chinese	F	25	Lower extremity weakness and movement disorder	Resting tremor, rigidity, movement disorder, and memory decline	Normal	Levodopa/effective	c.856delT	c.856delT	Gao et al. 2020 (28)
36	English	F	27	Dystonia	Dystonia, cognitive decline, anxiety, and depression	Cerebellar atrophy.	Levodopa/effective	c.956C>T	c.1061T>C	Magrinelli et al. 2022 (29)
37	English	F	29	Trembling and executive dysfunction	Resting tremor, movement disorder, rigidity, and mental disorder	Cerebellar atrophy.	Levodopa/effective	c.238G>A	c.1924A>G	Magrinelli et al. 2022 (29)
38	Indian	F	21	Trembling and mental and behavioral disorders	Resting tremor, movement disorder, rigidity, and mental disorder	Cerebellar atrophy.	Levodopa/effective	c.673C>T	c.2311G>A	Magrinelli et al. 2022 (29)
39	Indian	M	29	Trembling	Resting tremor, movement disorder, rigidity, and mental disorder	Cerebellar atrophy.	Levodopa/effective	c.1937C >T	c.1937C>T	Magrinelli et al. 2022 (29)
40	Indian	F	25	Trembling	Resting tremor, movement disorder, rigidity, and mental disorder	Cerebellar atrophy.	Levodopa/effective	c.2370T > G	c.1511C>T	Magrinelli et al. 2022 (29)
41	Indian	F	15	Depression and dysphoria	Anomalies of posture, cognitive decline, dysphoria, depression, mental disorder, and anxiety	Cerebellar atrophy.	Levodopa/effective	c.2222G>A	c.2222G>A	Magrinelli et al. 2022 (29)
42	Pakistani	F	23	Dysphoria	Dysarthria, cognitive decline, dysphoria, depression, urinary dysfunction, and anxiety	Cerebellar atrophy.	Levodopa/effective	c.2222G>A	c.2222G>A	Magrinelli et al. 2022 (29)
43	Pakistani	F	21	Dysphoria and depression	Cognitive decline, myoclonus, dysphoria, depression, and anxiety	Normal	Levodopa/effective	c.2222G>A	c.2222G>A	Magrinelli et al. 2022 (29)
44	German	M	22	Dyspraxia and gait instability	Dyspraxia, gait instability, dysarthria, and cognitive decline	Cerebellar atrophy.	Levodopa/effective	c.1021G>A	c.1898C>T	Magrinelli et al. 2022 (29)
45	Indian	M	21	Mental disorder and dysphoria	Spasm of eyelid, dysarthria, mental and behavioral disorders, and anxiety	Cerebellar atrophy.	Levodopa/effective	c.2222G>A	c.2222G>A	Magrinelli et al. 2022 (29)
46	Pakistani	M	31	Gait disorder, frequent falls	Myoclonus and cognitive decline	Cerebellar atrophy.	Levodopa/effective	c.2239C>T	c.2239C>T	Magrinelli et al. 2022 (29)
47	Hungarian	F	3	Gait instability and dysarthria	Gait instability, dysphagia, movement disorder, mental deterioration, trembling, dysphoria, and anxiety	Cerebellar atrophy.	NA	c.1864C>T	c.1798C>T	Toth-Bencsik et al. 2021 (30)
48	Chinese	M	29	Dyspraxia, gait disorder, and rigidity	Constipation, dreaminess, dysphoria, olfactory impairment, gait disorder, dyspraxia, and rigidity	Normal	Levodopa/effective	c.991G>T	c.1A>G	Chen et al. 2022 (31)
49	Chinese	M	20	Movement disorder	Anomalies of posture, gait disorder, trembling, movement disorder, rigidity, dysarthria, and cognitive decline	Cerebellar atrophy.	Levodopa/effective	c.991G>T	c.1117G>A	Cheng et al. 2022 (2)
50	Chinese	M	29	Gait disorder	Anomalies of posture, gait disorder, resting tremor, movement disorder, rigidity, and dysarthria	Cerebellar atrophy.	Levodopa/effective	c.991G>T	c.1915delG	Cheng et al. 2022 (2)
51	Chinese	M	31	Dysarthria	Anomalies of posture, gait disorder, resting tremor, rigidity, dysarthria, cognitive decline, and gait disorder	Cerebellar atrophy.	Levodopa/effective	c.967G>A	c.1450G>T	Cheng et al. 2022 (2)
52	Chinese	F	35	Movement disorder	Anomalies of posture, gait disorder, resting tremor, movement disorder, rigidity, dysarthria, and cognitive decline	Cerebellar atrophy.	NA	c.991G>T	c.1631T>C	Cheng et al. 2022 (2)
53	Chinese	F	6	Gait disorder	Anomalies of posture, gait disorder, trembling, and cognitive decline	Cerebellar atrophy.	NA	c.991G>T	c.1427+2T>A	Cheng et al. 2022 (2)
54	Chinese	M	15	Gait disorder	Anomalies of posture, gait disorder, trembling, movement disorder, rigidity, dysarthria, and cognitive decline	Cerebellar atrophy.	NA	c.1077G>A	c.1670C>T	Cheng et al. 2022 (2)
55	Chinese	M	24	Gait instability, movement disorder, and reduced expression	Movement disorder, gait instability, reduced expression, and cognitive decline	Cerebellar atrophy.	Levodopa/effective	c.991G>T	c.1427 + 1G>A	Lili Gao et al. 2022 (3)
56	Chinese	F	29	Gait instability, lower extremity weakness, and rigidity	Rigidity, movement disorder, frequent falls, cognitive decline, dysarthria, ocular paralysis, lower extremity weakness, and gait disorder	Cerebellar atrophy.	Levodopa/effective	c.967G>A	c.116G>A	Wan et al. 2022 (32)
57	Chinese	M	14	Gait instability	Gait instability, dysarthria, and mental disorder	Cerebellar atrophy.	Levodopa/Invalid	c.2120dupA	c.2071C>G	Wan et al. 2022 (32)
58	Chinese	F	3	Lower extremity weakness	Lower extremity weakness, rigidity, and mental disorder	Cerebellar atrophy.	NA	c.238G>A	c.1534T>A	Wan et al. 2022 (32)
59	Chinese	M	28	Muscle rigidity and resting tremor	Rigidity, resting tremor, movement disorder, and gait disorder	Cerebellar atrophy.	Levodopa/effective	c.991G>T	c.1054_1058delinsCTGGCCAGGAG	Ma et al. 2022 (33)
60	Chinese	M	30	Gait disorder, movement disorder, and dysarthria	Gait disorder, movement disorder, dysarthria, and frequent falls	Cerebellar atrophy.	Levodopa/effective	c.967G>A	c.1450G>T	Ma et al. 2022 (33)
61	Chinese	F	31	Gait instability, movement disorder, and trembling	Resting tremor, rigidity, movement disorder, and frequent falls	Cerebellar atrophy.	Levodopa/Invalid	c.991G>T	c.1771C>T	Ma et al. 2022 (33)
62	Chinese	M	11	Gait instability, movement disorder, and slow development	Gait disorder, dysphagia, slow development, severe dysarthria, and dystonia	Cerebellar atrophy.	Levodopa/effective	c.991G>T	c.1454G>A	This article

F, female; M, male; NA, not available.

By reviewing the literature, it was found that among the 62 probands, the male to female ratio was 2: 3, and the average onset age of male patients was 22.9 ± 8.7 years old. The average age of onset in women was 23.0 ± 8.5 years. There were 51 cases (82.2%) with movement disorder as the initial symptom, including 27 cases (43.5%) with gait disorder, 10 cases (16.1%) with gait instability, and 11 cases (17.7%) with limb trembling. There were 14 cases (22.6%) with depression, dysphoria, and other emotional instability as the initial symptoms. All probands were examined by cranial MRI, and 41 (66.1%) patients had cerebellar atrophy.

Among the 30 cases of national probands, the male to female ratio was 3:2, and the average onset age of male patients was 22.9 ± 8.9 years. The average age of onset in women was 22.9 ± 8.8 years. The most common mutation was c.991 G > T mutation in 21 families (70%), followed by c.967 G > A mutation in three families (10 %), c.1077 G > A mutation in three families (10%), and other mutations were rare. The most common clinical manifestations were movement disorder in 30 cases (100%), including gait disorder in 17 cases (56.7%), limb trembling in 19 cases (63.3%), and mental and behavioral disorders in 24 cases (80%). Among them, movement disorder was the most common symptom, with 28 cases (93.3%).

Among the 15 European and American probands, the ratio of male to female was 1:14. The most common clinical manifestations were movement disorder in 15 cases (100 %) and mental and behavioral disorders in 14 cases (93.3 %). Among them, mental and behavioral disorders were the most common symptoms, a total of 14 cases (93.3 %).

Of the 14 probands from the Middle East and western Asia, the ratio of male to female was 5: 9. The most common clinical symptoms were movement disorder (10 cases, 71.4%) and mental and behavioral disorders (13 cases, 92.9%). The most common symptoms were movement disorder (nine cases, 64.3%) and mental and behavioral disorders (seven cases, 50%).

Among the reported mutations, the most common mutation was c.991G > T in 21 families (33.9%), followed by c.2222G > A in eight families (12.9%), and other mutation types were rare. Among them, the c.991 G > T mutation only was found in Chinese, and the c.2222 G > A mutation was mainly distributed in the Middle East, western Asia and other countries. Among them, the ratio of male to female in the proband with c.991G > T mutation was 13:8. There were 15 cases (71%) of compound heterozygous mutations and six cases (29%) of homozygous mutations. The average age of onset of patients with compound heterozygous mutations was 23.1 ± 9.2 years old, and the most common initial symptom was movement disorder in seven cases (46.7%). There were 14 cases (93.3%) with dystonia and 10 cases (66.7%) with mental and behavioral disorders. The average age of patients with homozygous mutations was 32.5 ± 4.5 years old, and the initial symptoms were atypical, including one case of gait disorder (16.7%) and one case of movement disorder (16.7%). There were six cases (100%) of dystonia and three cases (50%) of mental and behavioral disorders. Among the probands with compound heterozygous mutations, there were 14 cases (93%) of cerebellar atrophy, and no cerebellar atrophy was found in the probands with homozygous mutations. The male to female ratio of the proband with c.2222G > A mutation was 1:7, all of which were homozygous mutations. The average age of onset was 23.3 ± 7.6 years. The most common initial symptoms were mental and behavioral disorders in seven cases (87.5%), including dysphoria, depression and other symptoms in 6 cases (75.0%). There were five cases (62.5%) of dystonia and eight cases (100%) of mental and behavioral disorders. A total of four probands had cerebellar atrophy (50%).

In addition, among all the probands, 9 (33%) patients with homozygous mutations had cerebellar atrophy, and 36 (85%) patients with compound heterozygous mutations had cerebellar atrophy.

## 4 Discussion

PLA2G6 gene is located in 22q13.1 region, about 6.0 Mb, containing 17 exons, encoding 85 ku cytosolic Ca2^+^ independent phospholipids A2 (iPLA2). There are two forms of iPLA2-A and iPLA2-β. The iPLA2-β enzyme is closely related to neurodegenerative diseases, and different mutation sites can lead to different degrees of changes in iPLA2-β enzyme. This leads to different clinical phenotypes of PLAN (31). The mutation types of this gene include missense mutation, truncation mutation and copy number variation, but the specific mechanism of this mutation is not clear (31). Previous studies have shown that the pathogenesis of EOP caused by PLA2G6 gene may be the loss of iPLA2 enzyme protein function caused by PLA2G6 gene mutation, which in turn causes phospholipid metabolism disorder of nerve cell membrane, intracellular iron deposition, lipid peroxidation, mitochondrial inner membrane damage, and Golgi morphological changes, eventually leading to a large number of apoptosis of dopaminergic neurons, decreased dopamine secretion, and the presence of Lewy bodies formed by misfolding and aggregation of α-synuclein in surviving neurons, leading to the occurrence of EOP (34, 35).

After reviewing the literature, this study showed that there were slightly more female patients with PLA2G6 gene-related EOP than male patients, and all of them had similar age of onset. The average age of onset was about 22 years old. The patients of EOP usually had gait disorder and movement disorder as the initial symptoms, but the resting tremor was relatively rare. As the disease progressed, it might be accompanied by symptoms such as rigidity, cognitive decline, mental and behavioral disorders (36–38). The majority of patients responded well to levodopa preparations, but the incidence of dyskinesia and symptom fluctuations reported in the literature was high and occurred earlier (34).

Based on retrospective analysis, it was found that the most common mutation in Chinese people was c.991G > T. One of the mutations reported in this study was also this variant. The mutation accounted for more than half of the Chinese pedigrees reported. It was further confirmed that the c.991G > T was the hot spot mutation of the PLA2G6 gene in China (2, 27), suggesting that this mutation had a founder effect in Chinese patients. The most common mutation reported abroad was c.2222G > A, which was mainly found in the Middle East and western Asia, including Arab, Saudi Arabia, India, Pakistan and other countries. The most common symptoms of c.991G > T mutation-related patients were movement disorder and gait disorder. Patients with c.2222G > A mutation usually had cognitive impairment, anxiety, depression, dysphoria, and other mental disorders, accompanied by a small amount of movement disorders. It was found that among the EOP probands caused by PLA2G6 gene, the probands with mental and behavioral disorders in Europe and America, western Asia and the Middle East were significantly higher than those in Chinese probands, which further confirmed the correlation between the clinical phenotype of EOP and different genotypes (29). However, Cheng et al. suggested that it might also be due to the complex phenotypic characteristics of Chinese patients, which could easily cover up symptoms such as myoclonus, cognitive decline and mental and behavioral disorders (2), suggesting that the evaluation of cognitive and mental disorders in EOP patients should be strengthened in clinical work.

In this study, 21 probands with c.991G > T mutation reported previously were further analyzed. It was found that patients with c.991G > T mutation usually had movement disorder, gait disorder and other symptoms as the first symptoms, followed by aggravation of symptoms and dystonia, resting tremor and other motor symptoms and non-motor symptoms. Among them, patients with c.991 G > T homozygous mutation occurred about 10 years later than those with compound heterozygous mutation. Furthermore, the initial symptoms were atypical and the clinical manifestations were milder. All of them were sensitive to levodopa treatment, which was consistent with previous studies (3, 11, 16). In addition, this study found that almost all of the probands with compound heterozygous mutations at this variant had cerebellar atrophy, while no cerebellar atrophy occurred in the six homozygous mutant probands, further suggesting that the clinical manifestations of patients with homozygous mutations at this variant were relatively mild. Previous *in vitro* cell experiments showed that c.991G > T mutant cells still retained 30% iPLA2β enzyme activity compared with wild-type cells, but the iPLA2β enzyme activity in H597fx69 cells expressing frameshift mutations only retained 6% (39). Because different mutation sites have different effects on iPLA2β enzyme activity, the reason for the difference between the two may be that another heterozygous mutation site outside the c.991 G > T mutation site has a greater effect on iPLA2β enzyme activity. The PLA2G6 protease activity of patients is higher than that of patients with heterozygous mutations, but more *in vitro* experiments of non-frameshift mutations are needed for further verification in the future. Therefore, we hypothesize that heterozygous and homozygous mutations in the PLA2G6 gene have different effects on the activity of iPLA2β enzyme, and the proportion of iPLA2β enzyme activity loss can partially explain that homozygous mutation probands have relatively benign clinical and neuroimaging phenotypes compared with heterozygous mutation probands (16).

This study also found that 64.8% of the probands showed brain atrophy on head MRI, but most studies showed that only a small number of EOP probands showed iron deposition on head MRI (31, 37, 40). From a pathological point of view, PLAN is characterized by the depletion of neurons in the cerebellar cortex, accompanied by astrocyte proliferation, axonal spheroids in the central and peripheral nervous system, and progressive brain iron deposition (2), cerebellar atrophy is the earliest sign on head MRI, while the signs of brain iron deposition in the basal ganglia often appear later. This may be the reason why MRI cerebellar atrophy signs are common and iron deposition signs are rare in EOP probands (2). Some researchers found that pro-inflammatory cytokines were significantly up-regulated, microglial activation, and reactive astrocyte proliferation were found in the pathological tissues of patients. Therefore, it is believed that inflammatory response is involved in the pathological process of cerebellar atrophy, and it is speculated that early anti-inflammatory treatment may help to delay the progression of cerebellar atrophy in patients with Parkinson's syndrome (41).

Another compound heterozygous mutation c.1454G > A in the PLA2G6 gene of the proband in this study has not been reported. Like most other EOP patients with PLA2G6 compound heterozygous mutations, the symptoms of PLA2G6 gene-related EOP in this patient were basically similar. The onset of the disease was 11 years old, with gait disorder, and the clinical manifestation was dystonia-Parkinson syndrome. The genetic test results of the proband's younger brother were consistent with those of the proband, and the age of onset was 11 years old. However, the clinical symptoms of the proband's younger brother were significantly lighter than those of the proband. The initial symptoms were anomalies of gait, and he could walk normally and take care of himself with the progression of the disease, and were not accompanied by symptoms such as dysphonia and dysphagia. It was speculated whether the proband had more susceptible genes than his younger brother, such as GBA, MAPT, SNCA, etc., leading to more severe clinical symptoms (42). In addition, different hormone levels could also affect the progression of Parkinson's disease (29). Considering that the living environment and habits of the proband and the proband's brother were roughly the same, this might also be one of the reasons for the clinical differences between the two.

Although the incidence of EOP is not high, the morbidity and mortality are very high (38), and most patients have a good response to treatment such as madopar 5–10 years after onset (3). This study also found that the vast majority of PLA2G6 gene-related EOP responds to levodopa treatment, but the delayed use of levodopa will increase the incidence of dyskinesia, and the switching period fluctuation is more obvious (33). Therefore, early diagnosis is of great significance for early initiation of anti-Parkinson therapy.

Because the sample size of most studies on PLA2G6 gene-related EOP in Chinese population is relatively small (3, 16, 27), this leads to limitations in the clinical and phenotypic comparison of different PLA2G6 gene mutation reviews in this study. The clinical and genetic characteristics of PLA2G6 gene-related EOP patients in China will be more clear in future multicenter large sample studies.

## 5 Conclusion

In conclusion, this study reported a case of early-onset parkinsonism caused by a novel variant of PLA2G6 gene and reviewed previous reports. This expands the genetic pedigree of the disease and increased clinicians' understanding of the clinical and genetic characteristics of early-onset parkinsonism.

## Data availability statement

The datasets presented in this article are not readily available because of ethical and privacy restrictions. Requests to access the datasets should be directed to the corresponding author.

## Ethics statement

The studies involving humans were approved by the Ethics Committee of Qujing First People's Hospital. The studies were conducted in accordance with the local legislation and institutional requirements. Written informed consent for participation in this study was provided by the participants' legal guardians/next of kin. Written informed consent was obtained from the individual(s), and minor(s)' legal guardian/next of kin, for the publication of any potentially identifiable images or data included in this article.

## Author contributions

DC: Conceptualization, Data curation, Formal analysis, Writing – original draft. HW: Conceptualization, Methodology, Supervision, Writing – review & editing. BH: Conceptualization, Methodology, Supervision, Writing – review & editing. WX: Data curation, Methodology, Writing – review & editing. KD: Conceptualization, Formal analysis, Funding acquisition, Investigation, Methodology, Software, Supervision, Validation, Writing – review & editing.
